# Controlling the electronic and physical coupling on dielectric thin films

**DOI:** 10.3762/bjnano.11.132

**Published:** 2020-10-01

**Authors:** Philipp Hurdax, Michael Hollerer, Larissa Egger, Georg Koller, Xiaosheng Yang, Anja Haags, Serguei Soubatch, Frank Stefan Tautz, Mathias Richter, Alexander Gottwald, Peter Puschnig, Martin Sterrer, Michael G Ramsey

**Affiliations:** 1 Institute of Physics, University of Graz, NAWI Graz, Universitätsplatz 5, 8010 Graz, Austria; 2 Peter Grünberg Institute (PGI-3), Forschungszentrum Jülich, 52425 Jülich, Germany; 3 Jülich Aachen Research Alliance (JARA), Fundamentals of Future Information Technology, 52425 Jülich, Germany; 4 Experimentalphysik IV A, RWTH Aachen University, 52074 Aachen, Germany; 5 Physikalisch-Technische Bundesanstalt (PTB), 10587 Berlin, Germany

**Keywords:** decoupling, integer charge transfer, organic films, *para*-sexiphenyl, thin dielectric film

## Abstract

Ultrathin dielectric/insulating films on metals are often used as decoupling layers to allow for the study of the electronic properties of adsorbed molecules without electronic interference from the underlying metal substrate. However, the presence of such decoupling layers may effectively change the electron donating properties of the substrate, for example, by lowering its work function and thus enhancing the charging of the molecular adsorbate layer through electron tunneling. Here, an experimental study of the charging of *para*-sexiphenyl (6P) on ultrathin MgO(100) films supported on Ag(100) is reported. By deliberately changing the work function of the MgO(100)/Ag(100) system, it is shown that the charge transfer (electronic coupling) into the 6P molecules can be controlled, and 6P monolayers with uncharged molecules (Schottky–Mott regime) and charged and uncharged molecules (Fermi level pinning regime) can be obtained. Furthermore, it was found that charge transfer and temperature strongly influence the orientation, conformation, and wetting behavior (physical coupling) of the 6P layers on the MgO(100) thin films.

## Introduction

Since the first scanning tunneling microscope (STM) imaging of the highest occupied molecular orbital (HOMO) and the lowest unoccupied molecular orbital (LUMO) of pentacene (5A) on NaCl/Cu(111) was performed [1], the concept of decoupling molecules from metal substrates with large bandgap dielectric films has become widely accepted. Although such systems have become a rich field of research, particularly in the scanning probe microscopy community, it is often forgotten that the wide bandgap insulating layer is not a sufficient condition for decoupling. Although it reduces wave function overlap with the substrate, it can in fact promote charge transfer via tunneling. The determining factor is the energy level alignment of the frontier orbitals of the adsorbate relative to the Fermi level (*E*_F_) of the underlying metal [2–3]. As dielectric films can significantly reduce the work function, principally due to Pauli repulsion (pushback) at the metal interface, adsorbates of sufficiently high electron affinity (EA) will become negatively charged via tunneling from the underlying metal. This was predicted by Pacchioni et al. [4–6] and either inferred or observed for adsorbates ranging from metal atoms [7–8] and small molecules [9–10] to larger π-conjugated molecules [11–13].

**Figure 1:**
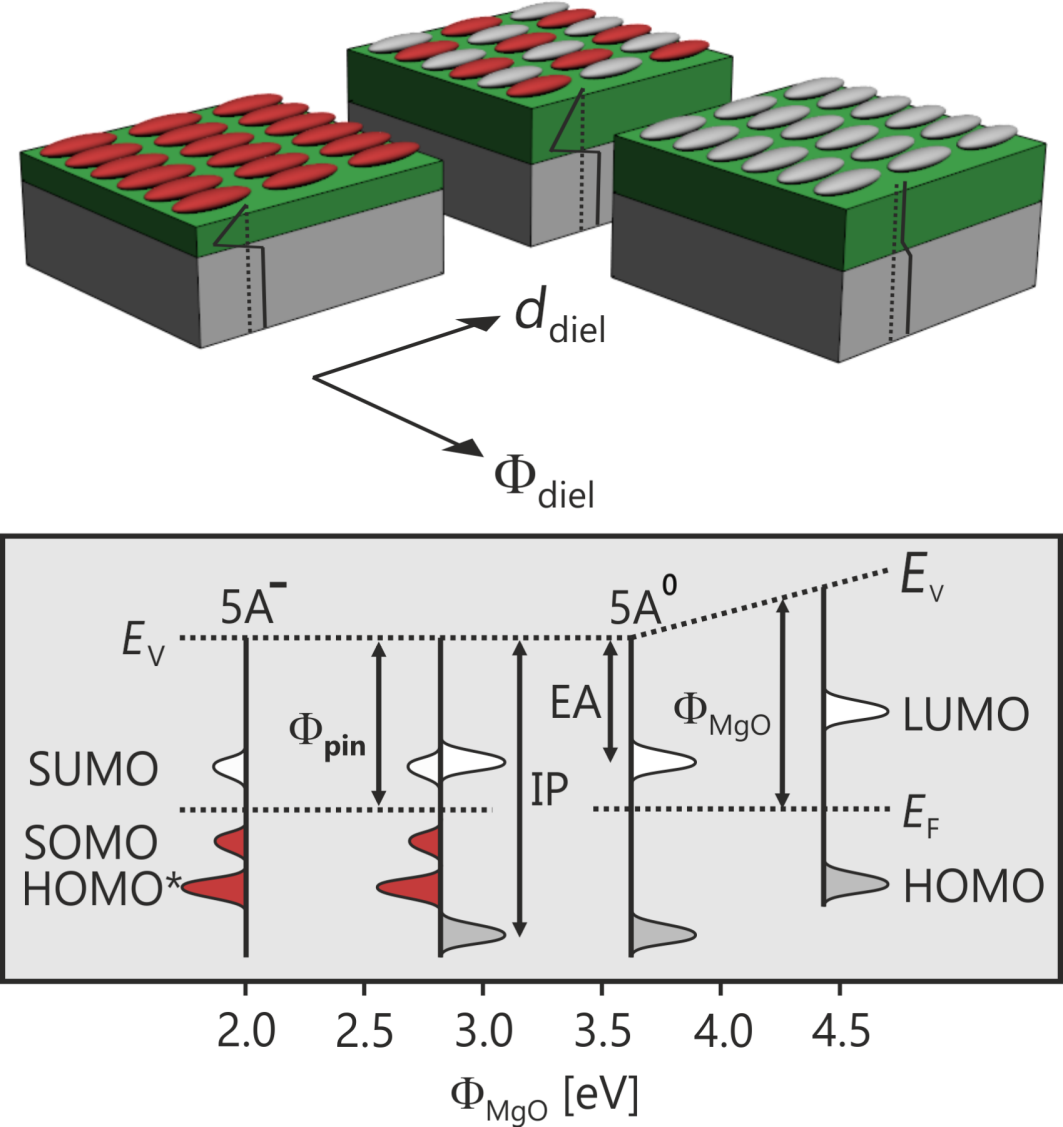
Molecular level schematic of integer charge transfer across dielectric films. Top: The ratio of integer charged (red) and neutral (gray) molecules is determined by the work function of the substrate after dielectric film growth (Φ_diel_) and the dielectric film thickness (*d*_diel_), according to the capacitor model. Bottom: Energy level alignment for charged 5A^−^ and neutral 5A^0^ on MgO(100)/Ag(100) from the Fermi level pinning to the vacuum level alignment regime. HOMO* refers to orbitals of charged molecules.

This phenomenon has been comprehensively analyzed for 5A on epitaxial MgO(100)/Ag(100), in which orbital-resolved STM and photoemission tomography (PT) have enabled the quantification of both the charge on individual molecules and the number of charged molecules in the 5A monolayer (ML) [14]. For 5A MLs on regularly grown epitaxial MgO(100) films, all molecules appear to be charged. Orbital-resolved STM reveals the LUMO both above and below the Fermi level, with a large gap between a singly occupied molecular orbital (SOMO) and a singly unoccupied molecular orbital (SUMO). In addition, PT confirms an integer charge transfer, which would be expected to result from tunneling [15]. However, using the ability to tune the MgO(100)/Ag(100) work function after MgO film growth (Φ_MgO_) by changing the composition at the dielectric–metal interface without changing its surface [16–19], it has been recently shown that two distinct adsorption regimes exist [20]. On films with a high Φ_MgO_, all molecules in the ML remain neutral, whereas on a low-Φ_MgO_ substrate, charge transfer is observed. In the former (the “vacuum level alignment” regime) the molecules are electronically decoupled, while in the latter, the electronic levels are tied to the Fermi level of the underlying Ag(100). The equilibration process for this “Fermi level pinning regime” has been realized by the proportion of charged and neutral molecules coexisting in the first monolayer, as schematically illustrated in Figure 1. In this work function regime, ranging from all the molecules being charged to no molecules being charged, the molecular ML is characterized by a constant pinning work function (Φ_pin_). The critical substrate work function (Φ_crit_) for the charge transfer is given by the condition Φ_MgO_ = Φ_pin_. On the macroscopic level, the simple relationships between the density of the integer charged molecules, the dielectric thickness (*d*_diel_), and the change in the work function upon adsorption of the molecules (ΔΦ = Φ_mol_ − Φ_MgO_, where Φ_mol_ is the final work function after adsorption of the molecules) is very well described by electrostatics with a simple capacitor model given by

[1] $$\Delta \Phi =\frac{\sigma \left(d_{\text{diel}}+d_{0}\right)}{\varepsilon_{0}\varepsilon_{\text{r}}}=\frac{\sigma d_{\text{cs}}}{\varepsilon_{0}\varepsilon_{\text{r}}},$$

where σ is the average charge density in the molecular film, ε_r_ is the dielectric constant of the thin film, and *d*_cs_ is the distance between the charge in the molecule and its image charge in the metal (i.e., the charge separation distance).

In this report, we demonstrate the robustness of the conclusions drawn from the 5A study by considering *para*-sexiphenyl (6P, C_36_H_26_). In contrast to 5A, 6P is geometrically more flexible, more specifically, it is nonplanar in the gas phase and has a relatively low electron affinity. On the pristine Ag(100) substrate, 6P simply physisorbs with no evidence of LUMO hybridization, which remains unoccupied [21]. It will be shown that the introduction of the dielectric interlayer can lead to integer charge transfer and electrostatic coupling to this otherwise passive metal surface. Unlike the 5A case, Φ_crit_ for the charge transfer is not equal to the Φ_pin_ for 6P. This is understood in terms of conformational changes in the 6P and the enhanced physical coupling to the substrate induced by the charge transfer. Finally, the charging effect on the thermal dynamics and the stability of the 6P monolayer are considered.

## Results and Discussion

### Ambivalent behavior of 6P on regularly prepared MgO(100)/Ag(100) thin films

**Figure 2:**
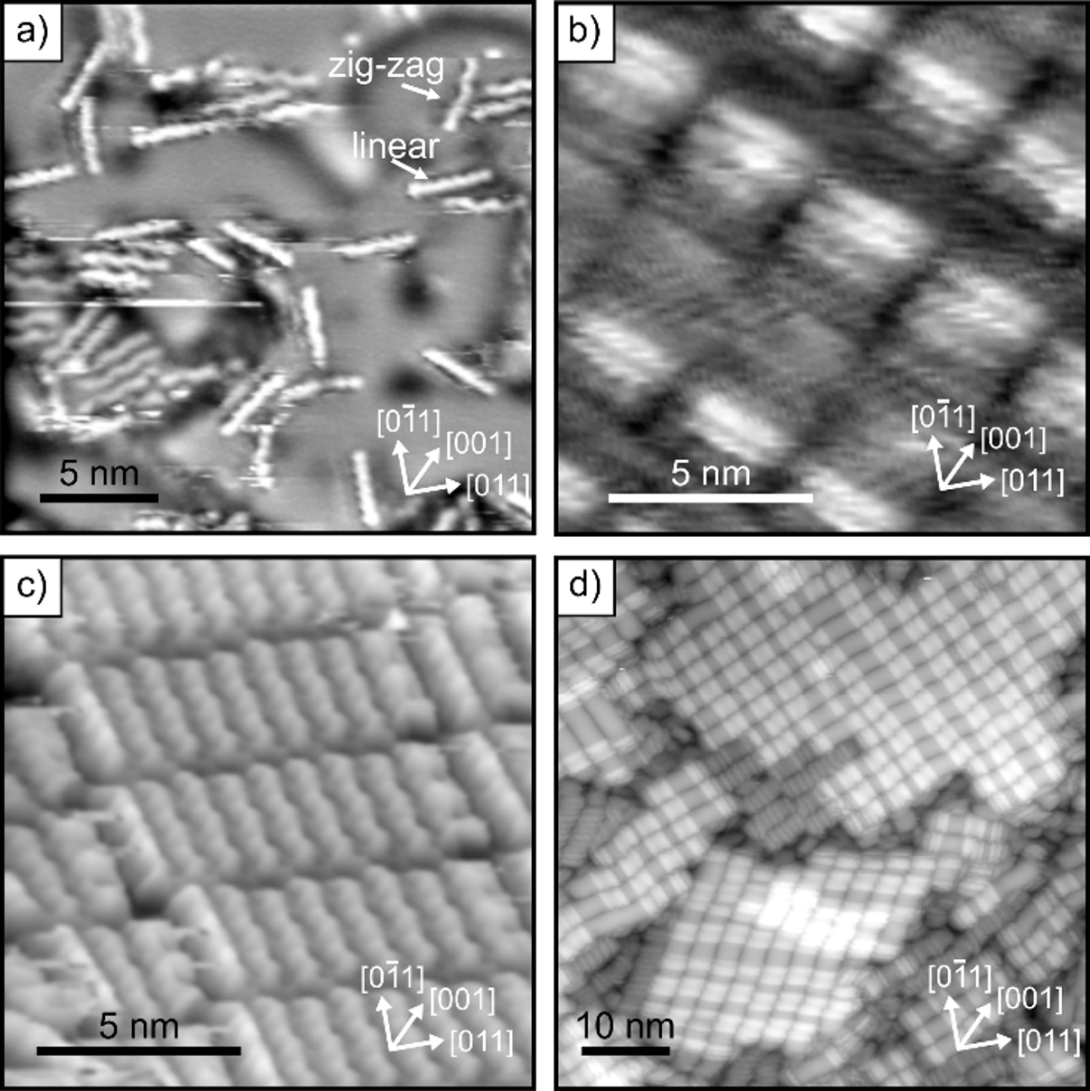
STM images of 6P deposited on (a,b) 2 ML MgO(100)/Ag(100) and (c,d) 3 ML MgO(100)/Ag(100). (a) Individual 6P molecules, *T*_dep,6P_ = −193 °C, size: 25 nm × 25 nm, *U*_b_ = +0.4 V, *i*_t_ = 25 pA; (b,c) 6P monolayer islands, *T*_dep,6P_ = 30 °C, size: 13 nm × 13 nm, *U*_b_ = +1.4 V, *i*_t_ = 16 pA (b) and *U*_b_ = −0.08 V, *i*_t_ = 140 pA (c). (d) Multilayer 6P islands, *T*_dep,6P_ = 30 °C, size: 60 nm × 60 nm, *U*_b_ = +1.7 V, *i*_t_ = 30 pA. All STM images were recorded at 80 K.

**Figure 3:**
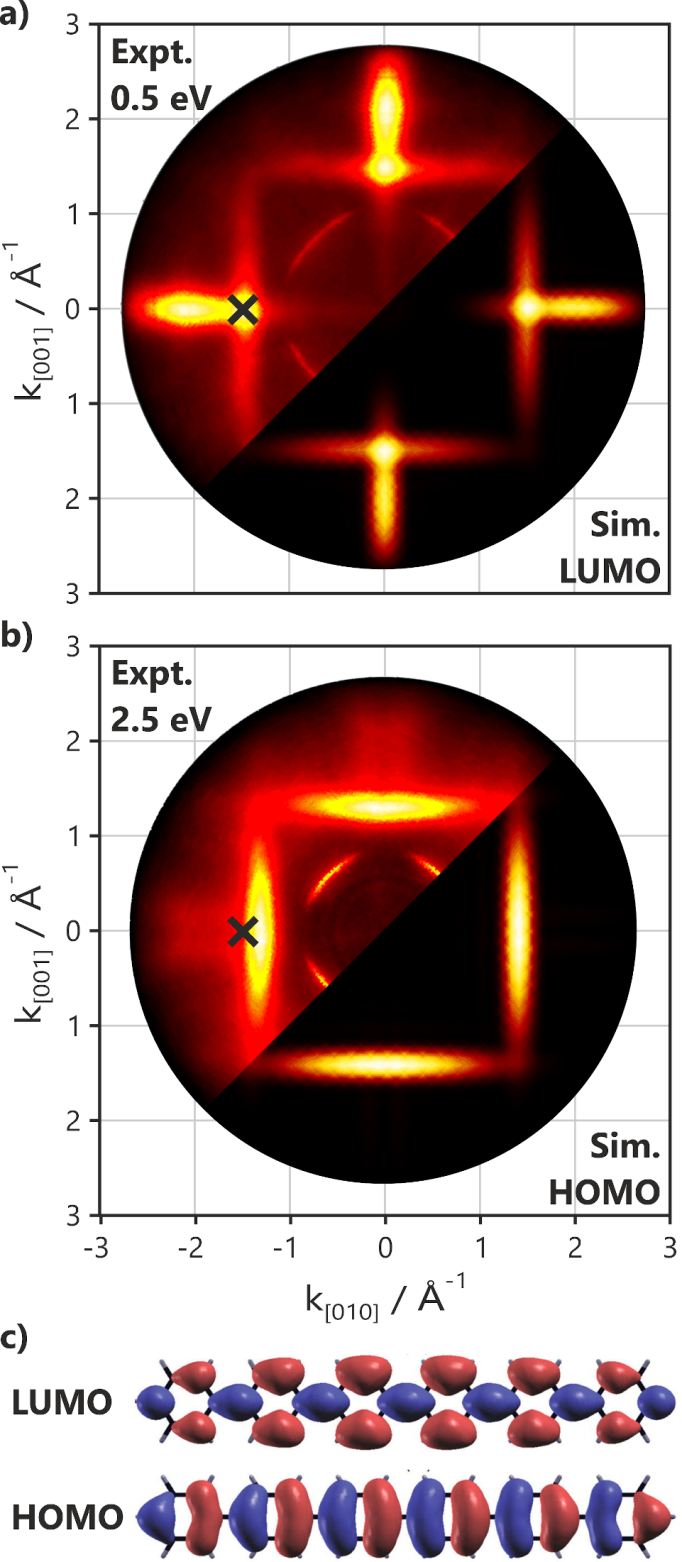
Photoemission momentum maps of 6P on MgO(100)/Ag(100) at energies of (a) 0.5 eV and (b) 2.5 eV below *E*_F_, measured with a photon energy of 35 eV and at an incidence angle of 40°. The experimental maps (top left of a and b) are shown in comparison to the maps calculated from the density functional theory orbitals (c) for two orthogonally oriented planar 6P molecules of the LUMO (bottom right of a) and the HOMO (bottom right of b). The geometry for the maximum LUMO intensity used in the following angle-resolved ultraviolet photoemission spectroscopy experiments is marked with crosses. Deviations from the simulations for isolated molecules are a result of MgO and Ag(100) sp-band emissions. Films of 6P were grown and measured at room temperature.

Figure 2 shows STM images of four 6P/MgO(100)/Ag(100) preparations for MgO films with a thickness of 2 ML (Figure 2a, Figure 2b) and 3 ML (Figure 2c, Figure 2d), and 6P coverage ranging from sub-ML (Figure 2a) and close-to-monolayer (Figure 2b, Figure 2c) to 2 ML (Figure 2d). STM shows 6P molecules arranged in ordered monolayer islands (Figure 2b, Figure 2c), with their long axes aligned parallel to each other and parallel to the substrate surface. This is typical for 6P on atomically clean and ordered substrates obtained from bulk oxides to metal substrates [21–25]. In the submonolayer coverage regime (Figure 2a), we find molecules with different orientations coexisting on the surface. Their long axes are aligned along the principal crystallographic directions [001]/[010] and [011]. In the monolayer regime, molecules align either along [001]/[010] (Figure 2b) or [011] (Figure 2c), while in Figure 2d, where multilayer islands of up to three layers high are present, the molecules in the domains are rotated by 18° with respect to [011]. Note that on some MgO preparations, severe island growth was observed, while on others it was less pronounced.

Generally, the molecules appear as rod-like features in STM. A pronounced six-lobe structure with a slight zig-zag appearance is seen in some of the molecules in Figure 2a. This structure is even more evident in the molecules forming the ordered island in Figure 2c. These may be associated with the six phenyl rings of the 6P molecule presenting a twisted conformation. In the gas phase, 6P naturally occurs with a torsional angle of 35° between its phenyl rings [26]. The observation of such a twist on MgO(100)/Ag(100) suggests that the interaction between the 6P molecules and MgO is very weak. In addition, next to the 6P molecules with a zig-zag appearance, there are other molecules with 6 lobes, but in a linear arrangement (Figure 2a). A possible explanation for this observation is the charging-induced planarization of 6P. For comparison, on the pristine Ag(100) substrate, planar 6P molecules are observed in the submonolayer regime despite the lack of any charge transfer, as evidenced by the absence of an emission from the LUMO in photoemission tomography [21]. This implies that the van der Waals interaction with the metal is sufficient to planarize the molecule. From the STM images shown here of 6P on the MgO(100)/Ag(100) films it is not possible to provide an unambiguous assignment of charged and uncharged molecules, since the orbital resolution has not been achieved in this system. However, the existence of two different 6P appearances (Figure 2a) and monolayer orientations (Figure 2b, Figure 2c) might be related to different charge states of the molecules on the surface.

The images of orbitals of molecules adsorbed on surfaces can also be obtained from the angular intensity distribution in valence band photoemission experiments via PT [27]. When the photoelectron emission angle is converted to momentum, the resulting momentum maps approximately reflect the square of the Fourier transform of the real space orbitals (i.e., the momentum maps image the orbital in the reciprocal space). PT experiments conducted on 6P monolayers on MgO(100)/Ag(100) revealed that films prepared under nominally identical conditions could yield apparently contradicting results. On some preparations, no molecular emissions were observed in the MgO bandgap, whereas on others, distinctive features appeared in the gap at 0.5 and 2.5 eV below the Fermi level. The momentum maps of these molecular emissions (Figure 3) can be unambiguously assigned to the orbitals and the geometry of the molecules from which they are emitted [28]. The experimental maps in Figure 3a and Figure 3b are in very good agreement with the simulations of the HOMO and LUMO for 6P having two orthogonal orientations. The observation of the LUMO emission clearly shows that charge transfer has occurred.

The azimuthal orientations of the molecules can be derived by comparing the orientations of the molecular emission patterns to the orientation of the emission pattern from the Ag(100) substrate or from the crystal surface unit cell inferred from low energy electron diffraction (LEED) experiments. This comparison shows that all charged molecules have their long axes aligned 45° with respect to the close-packed atomic rows of the substrate (i.e., along the [001]/[010] azimuths) in agreement with the orientation of the molecules obtained via STM in Figure 2b.

Significantly, the theoretical maps shown in Figure 3 are for flat lying planar molecules. The photoemission tomography technique is very sensitive to any out-of-plane tilt or twist in 6P [29–32]. A nonzero torsional angle leads to orbital periodicity doubling in the real space and orbital periodicity halving in the reciprocal space [23]. It can therefore be concluded that, while neutral molecules can have a twist in different orientations on the MgO surface, the molecules that have experienced charge transfer are planarized. Therefore, they lose the 35° torsional angle between their phenyl rings and align exclusively along [001]/[010]. It should be noted that the occupation of the LUMO is predicted to reduce the torsional angle in the molecule, given that it has a bonding character with respect to the phenyl rings (see Figure 3c) [33]. However, this is not a sufficient condition for planarization. Density functional theory (DFT) calculations for the isolated molecule indicate that the torsional angle changes from 35 to only 20° upon the formation of the anion. Presumably, the electrostatic interaction of the charged molecules with the substrate completes the planarization.

In summary these results suggest that MgO films prepared under nominally identical conditions can result in either charged or uncharged molecules. While uncharged molecules retain the torsional angle between their phenyl rings, the charge transfer is accompanied by a change in the orientation of the molecules and they become planar. In the following subsection, the critical role of the work function for charging and its influence on the surface wetting capability of the molecules will be highlighted.

### Work function control of electronic coupling

**Figure 4:**
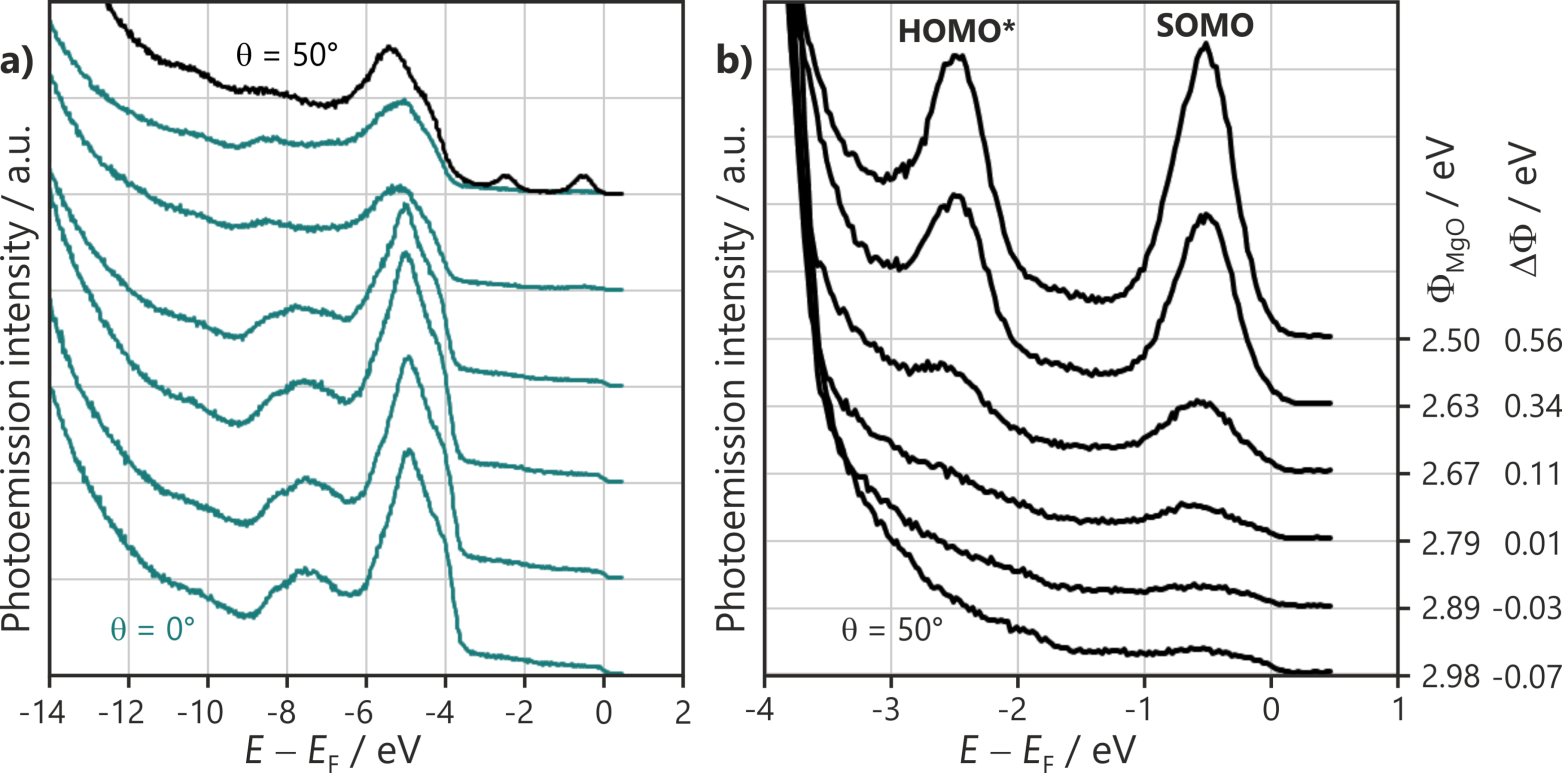
He I ARUPS spectra for different preparations with the same saturation exposure of 6P (4 Å) on the same 2 ML MgO film tuned to different work functions. Φ_MgO_ values and the change in Φ induced on 6P exposure (ΔΦ) are given on the right. (a) Wide scans at normal emission (θ = 0°) emphasizing the MgO emission region. The black curve shows the ARUPS spectrum measured at θ = 50° in the [001] azimuth, where the 6P HOMO* and SOMO emissions in the MgO bandgap region are prominent. (b) Magnification of the HOMO* and SOMO emissions in the MgO bandgap at θ = 50°.

Figure 4 displays angle-resolved ultraviolet photoemission spectroscopy (ARUPS) scans after the 6P molecules were submitted to the same saturation exposure on the same 2 ML MgO(100) film. The initial work function, Φ_MgO_, was tuned to different values. Figure 4a shows wide scans in normal emission, where the MgO valence band dominates at an energy between 9 and 4 eV below *E*_F_.

**Figure 5:**
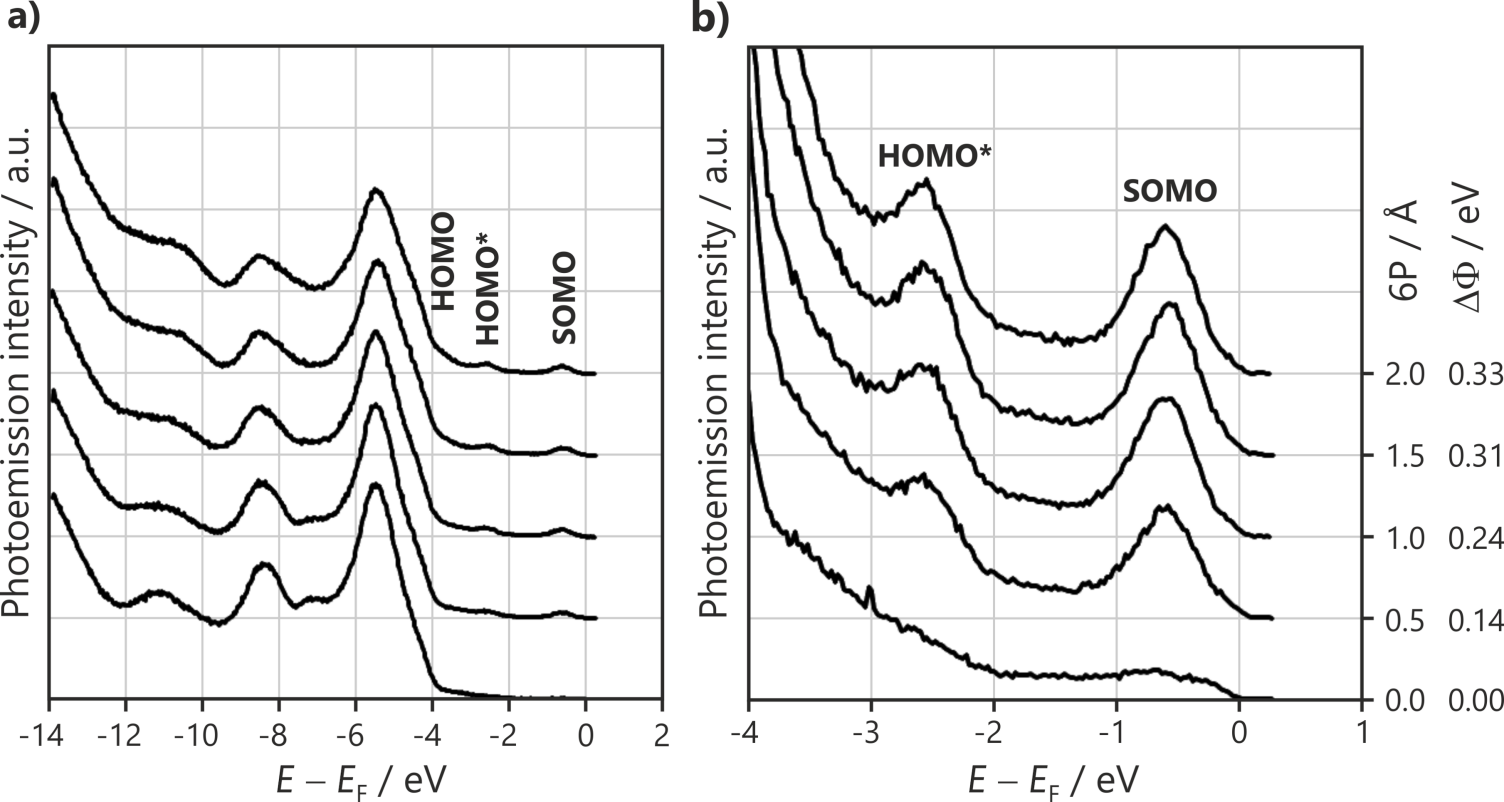
He I ARUPS spectra for a 6P dosing series on 2 ML MgO(100)/Ag(100) (Φ_MgO_ = 2.58 eV) recorded at a takeoff angle of 50° in the [001] azimuth. (a) Full scan and (b) magnification of the molecular emissions in the MgO bandgap. 6P exposure and change in the work function upon deposition are indicated on the right.

For Φ_MgO_ greater than 2.8 eV, there is only a minor decrease in the work function (<0.1 eV) and only a small attenuation in the MgO emissions of approximately 15% compared to the clean substrate, suggesting that only a small fraction of the surface is covered with molecules and 3D islands are formed. For Φ_MgO_ below 2.8 eV, the results change completely. They show the onset of significant substrate attenuation, changes in the spectral lineshape, and an increase in the work function upon 6P deposition. The attenuation behavior suggests that the molecules fully wet the surface only for Φ_MgO_ below 2.65 eV, where a 60% reduction in the MgO intensity is observed. The increase of the work function indicates a charge transfer to 6P. The charge transfer is observed directly by the presence of a LUMO emission seen at high emission angles in Figure 4a. Magnified scans of the MgO bandgap region in the geometry of maximum LUMO emission intensity, indicated by the crosses in Figure 3, are displayed in Figure 4b. For Φ_MgO_ greater than 2.8 eV, no significant orbital emissions are found in the gap, implying that no charged molecules are present on the surface. For Φ_MgO_ below 2.8 eV, molecular emissions arise at 2.52 and 0.55 eV with respect to *E*_F_. Their photoemission energies and angular distributions agree with the photoemission tomography of the HOMO and LUMO of the planar 6P seen in Figure 3. Note that the emissions in the MgO bandgap are due to the orbitals of charged molecules and will be referred to as HOMO* and SOMO henceforth. Neutral molecules are difficult to observe directly via ARUPS, as their HOMO emission lies below the gap in the region of intense MgO emissions (Figure 5a). The HOMO* and SOMO intensities increase with decreasing Φ_MgO_, indicating an increase in the number of charged molecules. Thus, Φ_MgO_ = 2.8 eV is the critical work function value, below which charge transfer into 6P molecules occurs. The extreme sensitivity to Φ_MgO_ shown in Figure 4 explains the apparently ambivalent results obtained from STM and photoemission tomography in the previous section, as the accepted procedure for growing epitaxial MgO(100) films on Ag(100) [19,34] produces a scatter of the Φ_MgO_ values on either side of the critical work function (Φ_crit_ = 2.8 eV).

Figure 5 shows the evolution of the ARUPS spectra with an increasing 6P dose on an MgO(100) film with Φ_MgO_ = 2.58 eV (i.e., below Φ_crit_). In the MgO valence band region (Figure 5a), gradual changes can be observed. In the MgO bandgap region (magnified in Figure 5b), the intensities of the HOMO* and SOMO increase and reach their maximum value at a 6P dose between 1 and 1.5 Å. The increase in the molecular emission features is accompanied by an increase in the work function (ΔΦ, values listed on the right). The dose corresponding to a full 6P monolayer is estimated to be approximately 2.8 Å from the attenuation behavior of the valence band of the underlying MgO. The fact that the molecular emissions and Φ saturate well before the completion of the ML suggests that a significant number of neutral molecules are also present in the ML on this specific MgO film. A close inspection of Figure 5a shows that the HOMO of the neutral molecules can be seen at ≈3.6 eV.

In previous research, the charge transfer to 5A on MgO(100)/Ag(100) was quantified by comparing ARUPS data to predictions made by PT, yielding a LUMO occupation of 1 electron [14]. Following the same approach for 6P, the ratio between the HOMO* and the SOMO maximum intensity values is predicted to be 0.57 for a singly occupied LUMO. The various 6P preparations yielded intensity ratio values ranging from 0.60–0.75, suggesting a LUMO occupation of 1.0 to 1.3 electrons. This is consistent with single integer charge transfer, expected for tunneling, when uncertainties in the theoretical description of the photoemission intensities of the simple plane-wave final-state approximation and in the experimental determination of the peak areas are considered.

### The capacitor model and the interlayer dielectric constant

**Figure 6:**
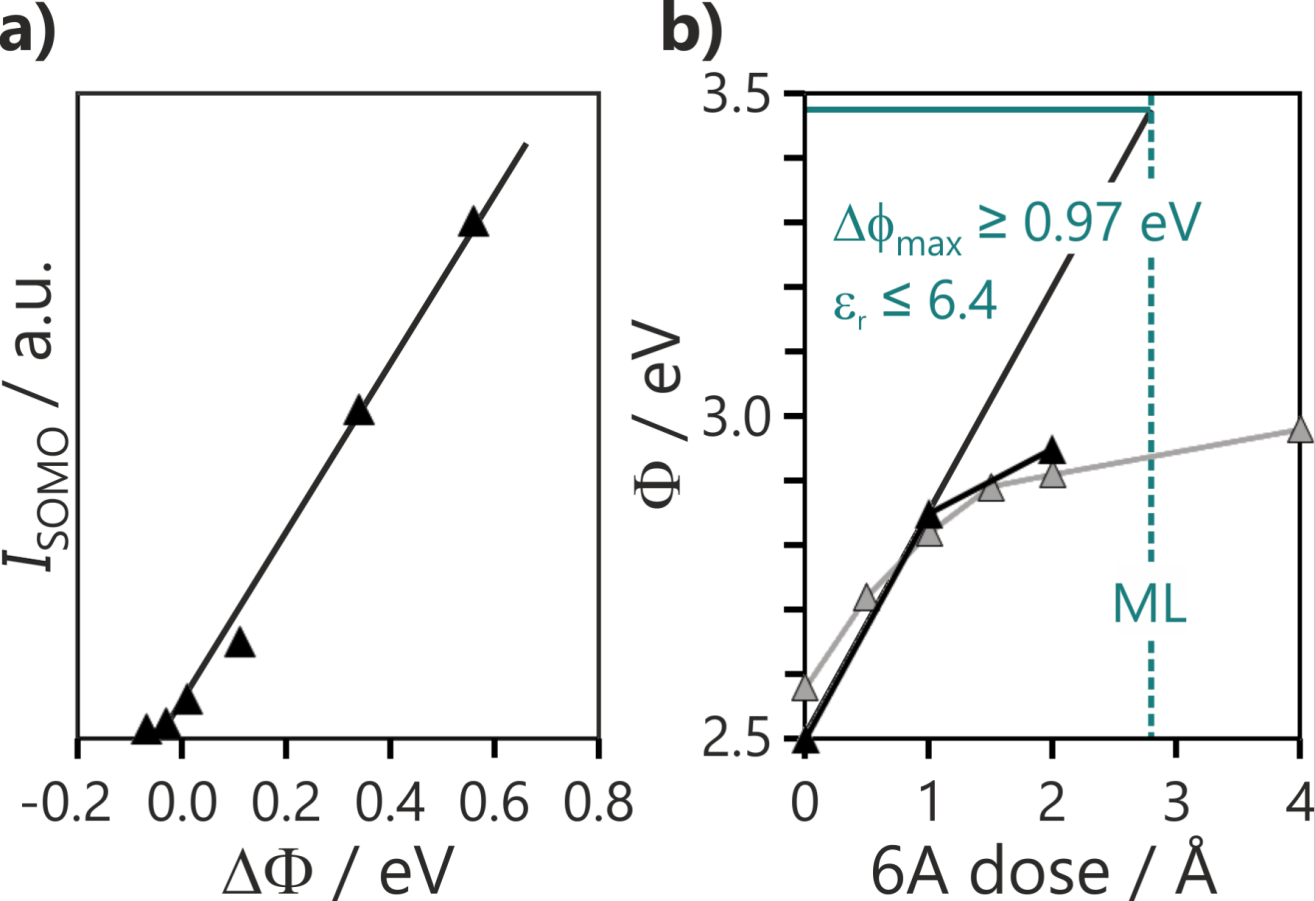
(a) Relation between integrated SOMO intensity and work function change induced by molecule adsorption on MgO(100)/Ag(100) (ΔΦ) after tuning Φ_MgO_ to slightly different values. (b) Work function versus 6P dose for two different initial work functions. The dose corresponding to a full ML is indicated by the dashed line.

The behavior observed in the ARUPS series of Figure 4 and Figure 5 is consistent with the predictions from the capacitor model expressed in Equation 1. Figure 6a depicts the relationship between the SOMO intensity (*I*_SOMO_) and the work function change induced by molecule adsorption (ΔΦ), obtained from the series of 6P saturation doses on MgO films with different Φ_MgO_ values. The linear proportionality between *I*_SOMO_ and ΔΦ agrees with the capacitor model [20], considering that *I*_SOMO_ reflects the number of integer charged molecules in the monolayer and thus the charge density (σ) on the surface.

For the capacitor model to be predictive and quantitative for ultrathin decoupling layers, a realistic value for their dielectric constant (ε_r_) is required. This can be estimated from a molecular dosing series, such as in Figure 5. Figure 6b shows the increase of Φ with an increasing 6P dose for two different MgO films with Φ_MgO_ < Φ_crit_ values. Initially, Φ increases rapidly with 6P exposure since all molecules become charged. The curve then flattens out as Φ approaches Φ_pin_ and additional molecules remain neutral. This is reached at an exposure of ≈1.4 Å, which is half of the amount required to complete the ML. It can be concluded that on these films there is a 50/50 mix of charged and neutral molecules in the completed monolayer.

Assuming all molecules are charged at low exposure, the ε_r_ value can be estimated from the initial slope (ΔΦ/σ) in Figure 6b, according to:

[2] $$\varepsilon_{\text{r}}=\frac{\sigma }{\Delta \Phi }\frac{d_{\text{cs}}}{\varepsilon_{0}}$$

In order to determine the charge density, the knowledge of the density of charged molecules in the monolayer is required. In the case of pentacene, this could be reliably measured with STM, as 5A monolayers with all molecules charged could be produced. The LEED image for 6P films on MgO(100)/Ag(100) with a high proportion of charged molecules showed faint superstructure spots, which suggest a later molecular spacing of 6.6 Å. The van der Waals dimensions of 6P (6.7 × 27.2 Å) [26] are close to the unit cell (6.35 × 27.47 Å) which is commensurate with MgO(100). The area of this unit cell corresponds to a molecular footprint of 173 Å^2^ and gives an upper bound of 6.4 for ε_r_. The ordered structure in the STM image of Figure 2b, where molecules do not show a zig-zag structure and are oriented along [11] (like the charged molecules in PT), is consistent with this lateral spacing. However, this structure suggests a slightly longer length of 31 Å for the long unit cell vector. This value yields a molecular area of 197 Å^2^ and an ε_r_ of 5.7. This estimate is in agreement with the values for ε_r_ (between 5 and 6) obtained for 5A/MgO(100)/Ag(100) using two different approaches and MgO thickness values ranging from 2 to 8 ML [20]. This suggests that the effective ε_r_ for MgO thin films is significantly smaller than the bulk value of 9.9 [35].

### Critical and pinning work functions

**Figure 7:**
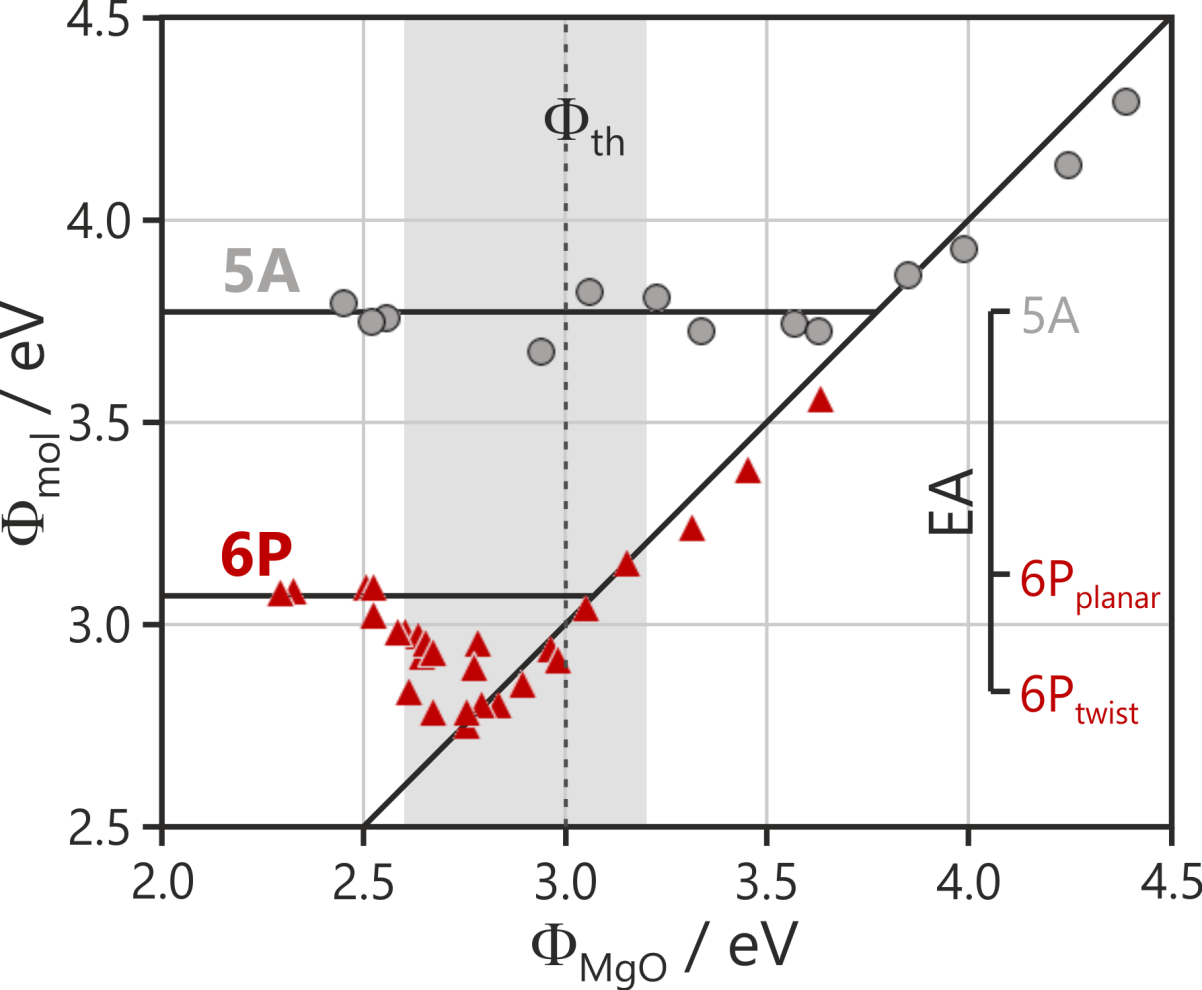
The work function after the deposition of a saturating dose of 5A (grey) and 6P (red) (Φ_mol_) as a function of the initial work function of a clean MgO(100)/Ag(100) substrate (Φ_MgO_). The line with a slope of unity expresses the Schottky–Mott limit of the vacuum level alignment (Φ_mol_ = Φ_MgO_). The horizontal lines correspond to the pinning work functions of 5A and 6P on 2 ML MgO films on Ag(100). The dashed line indicates the theoretical work function for a stoichiometric MgO–Ag(100) interface [36], and the shaded gray area indicates the most typical range of work functions obtained with MgO film growth under nominally identical conditions. The inserted scale shows the theoretical gas-phase electron affinities for 5A, twisted 6P, and planar 6P and it has been shifted to align the EA of 5A with the measured pinning work function for 5A.

The rather abrupt changes over a narrow work function range observed for both the charge transfer and wetting behavior for 6P are in contrast with the 5A system. In the latter, the number of charged molecules increases gradually with Φ_MgO_ below the critical work function, and a wetting ML is always present, whether or not the charge transfer occurs. Due to the critical role of the work function for charge transfer, it is convenient to plot the work function after saturating the surface with molecules (Φ_mol_) as a function of Φ_MgO_. This is shown in Figure 7 for both 5A and 6P on 2 ML MgO(100)/Ag(100). For the 5A case, the regimes of vacuum level alignment and Fermi level pinning are clearly expressed: In the vacuum level alignment regime, there is no charge transfer and the final work function closely follows the Schottky–Mott rule in which Φ_mol_ = Φ_MgO_ (the 45° line in Figure 7), albeit with a slight reduction in Φ due to Pauli repulsion (pushback). The Fermi level pinning is characterized by a constant final work function, Φ_pin_, and an increase in the number of charged molecules with decreasing Φ_MgO_. The number of charged molecules is determined by the potential needed to raise Φ to Φ_pin_ values, whereupon no further charge transfer can occur. It is quite intuitive that Φ_pin_ equals the critical work function for the onset of charge transfer, Φ_crit_.

However, surprisingly, this is not the case for 6P as shown in red in Figure 7. For high Φ_MgO_ values, again Φ_mol_ is essentially equal to Φ_MgO_. The critical work function for the charge transfer (i.e., the point where Φ_mol_ increases above the 45° line) is approximately 2.8 eV. This is significantly lower than the value for 5A, which is expected due to the lower EA of 6P (theoretical values of 2.39 eV and 1.45 eV, respectively, using the B3LYP functional). However, unlike for 5A, as Φ_MgO_ decreases further, Φ_mol_ does not remain constant but increases above Φ_crit_. This reflects the rapid increase in the number of charged molecules observed in Figure 4. Only for Φ_MgO_ ≤ 2.5 eV the final work function adopts a constant value of 3.07 eV, which is the pinning work function of 6P on 2 ML MgO(100)/Ag(100). Thus, for 6P, Φ_pin_ is 0.3 eV higher than Φ_crit_.

We suggest that this difference is related to the conformational change of 6P upon charging. Φ_crit_ derives from the electron affinity of the neutral, twisted molecule, while Φ_pin_ derives from the EA of the planar molecule. The planarization of the molecule will lead to greater orbital overlap between the phenyl rings. This will cause an increase in the energy spread of the π bands, resulting in a decrease in the ionization potential and an increase in the electron affinity. Gas phase DFT calculations show that the EA of planar 6P (1.74 eV) is indeed 0.3 eV higher than the EA of twisted 6P (1.45 eV). This is in close agreement with the measured difference between Φ_pin_ and Φ_crit_ seen in Figure 7, while only a negligible difference in molecular surface dipole is expected between twisted and planar molecules [37]. It should be noted that this alone cannot explain that, depending on the initial work function, either Φ_crit_ or Φ_pin_ is the decisive work function for charge transfer.

**Figure 8:**
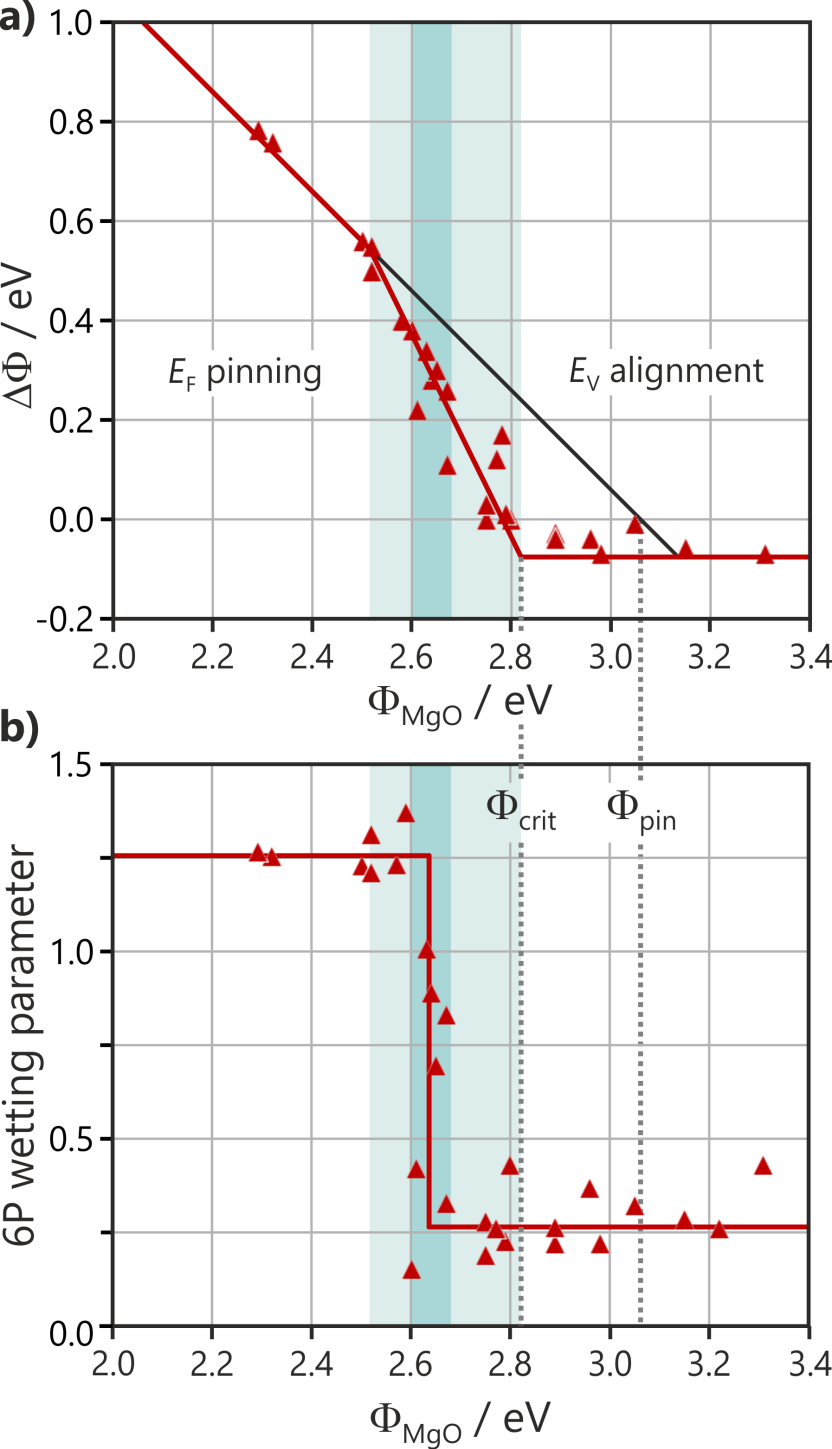
Changes in the charge transfer and wetting behavior of 6P on MgO(100)/Ag(100) as a function of Φ_MgO_. (a) Work function change induced by 6P as an indicator of charge transfer. The black 45° line corresponds to a constant final work function and expresses the behavior when Φ_pin_ = Φ_crit_. (b) Wetting parameter as an indicator of the fraction of the substrate covered by 6P.

In order to better understand the transition between the two regimes, an alternative representation of the data from Figure 7 is given in Figure 8a. In the latter, the work function change upon 6P deposition as a function of the substrate work function, for various 6P films on a number of different 2 ML MgO films is plotted. This is displayed in comparison to the 6P surface coverage, which is expressed by a wetting parameter in Figure 8b. The wetting parameter reflects the combined effects of the intensity of all molecular emissions and the suppression of substrate emissions (see Experimental section). The small wetting parameter in the vacuum level alignment regime confirms that molecules do not wet the surface, indicating a low molecule–substrate interaction and 3D island formation.

For Φ_MgO_ values between Φ_crit_ and 2.52 eV, Figure 8a exhibits a steep rise in ΔΦ and, therefore, in the number of charged molecules. However, the wetting indicated in Figure 8b remains low, until Φ_MgO_ reaches approximately 2.64 eV, where an abrupt discontinuous transition to high wetting occurs. We propose that the critical factor for this phase transition is the density of charged molecules present on the surface. The planarization and electrostatic interaction of the charged molecules with the substrate can be expected to reduce the mobility of the charged molecules. These charged molecules will in turn restrict the free motion of the neutral molecules and, at a critical density, immobilize and reorient them. This wetting transition occurs when 30% of the molecules are charged, as estimated from ΔΦ in the capacitor model, while the final Φ_pin_ is established after 60% of the molecules are charged.

### The effect of charge transfer and physical coupling on thermal stability

**Figure 9:**
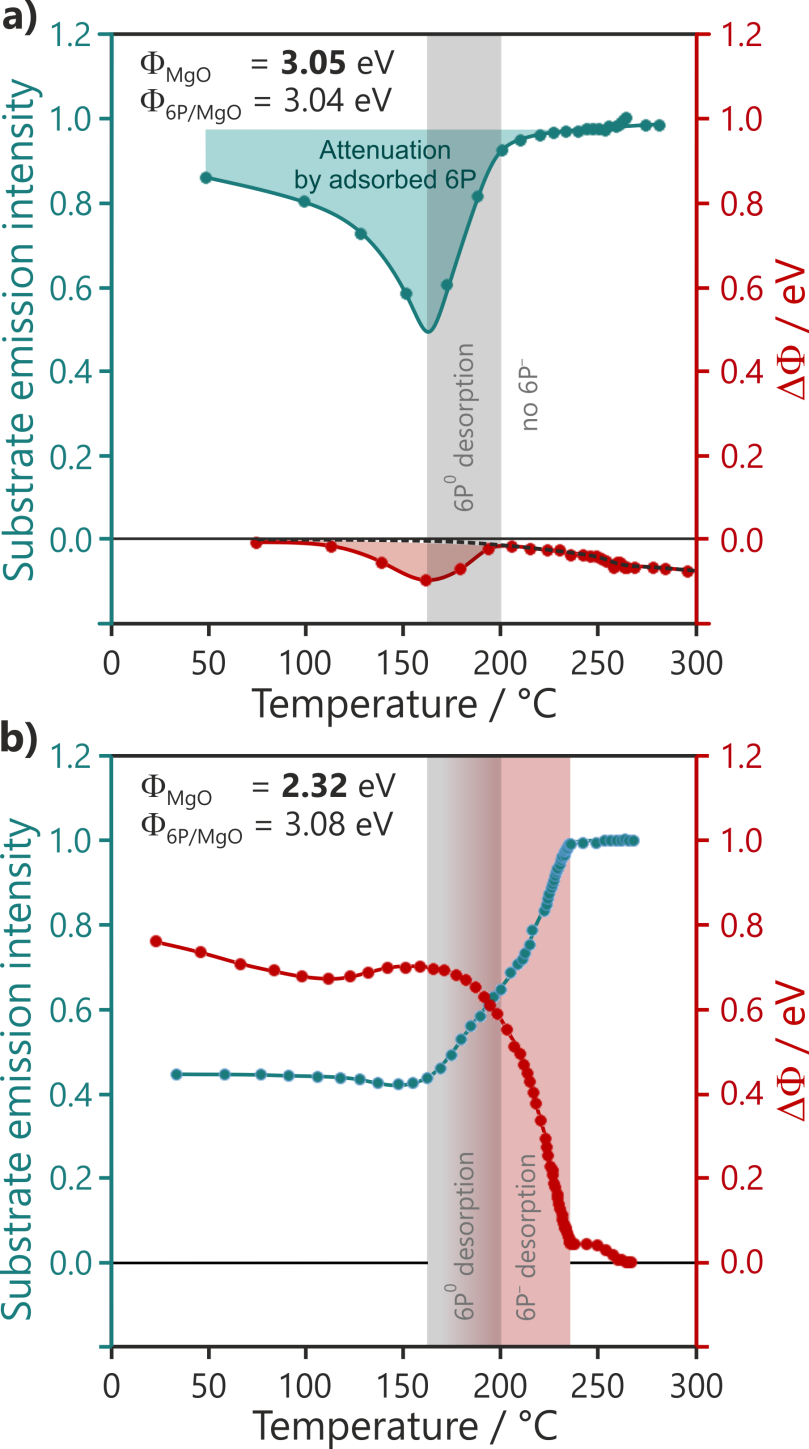
Thermal stability of 6P films (4 Å) on 2 ML of MgO with work functions (a) above and (b) below the critical work function. The blue curves depict the normalized photoemission intensity of the MgO valence band relative to the intensity after 6P film desorption. The red curves depict the change in work function relative to the pristine MgO film work functions, Φ_MgO_.

We now turn to the role of charge transfer on the physical coupling by considering the thermal stability of the molecular film. This is considered in Figure 9a and Figure 9b for a 6P deposit of 4 Å on two 2 ML thick MgO films for Φ_MgO_ values above (Figure 9a) and below (Figure 9b) the critical work function for charge transfer. The graphs show the intensity of the substrate photoemission signal (MgO valence band peak intensity in normal emission) and the response of the work function while increasing the temperature. For the high Φ_MgO_ film case, where the ARUPS results show no charged molecules on the surface, the temperature increase leads first to an apparently counterintuitive decrease in substrate intensity until the temperature reaches approximately 160 °C. This is followed by an abrupt increase in intensity until the temperature reaches 200 °C. At that point, since the intensity has almost returned to its value prior to the 6P deposition, all the molecules can be considered to be desorbed from the surface. This behavior is mirrored in the work function, which first decreases and then, at 160 °C, increases, returning to its original value by 200 °C. The initial substrate intensity suggests that, at room temperature, only approximately 20% of the surface is covered by molecules, implying that 3D islanding occurs. The temperature increase liberates molecules from the islands and allows them to wet the MgO surface. Right before their desorption at 160 °C, the attenuation of the substrate emission signal suggests a complete surface coverage. The temperature-induced wetting of the surface with neutral molecules is reflected in the pushback-induced lowering of Φ by ≈−0.1 eV.

The desorption behavior on the low Φ_MgO_ film, with up to 80% of the molecules charged according to the capacitor model (Figure 9b), is quite different. The initial substrate attenuation confirms that the molecules wet the surface at room temperature. There is no significant change in this attenuation until the temperature reaches 160 °C. At that point, the substrate intensity increases as the molecules begin to desorb from the surface. From Figure 9a, we already know that this is the desorption temperature of neutral molecules. However, on this MgO film, desorption continues well beyond 200 °C, and the substrate intensity returns to the value corresponding to the clean substrate at 230 °C. It stands to reason that this further increase in the substrate intensity between 200 and 230 °C is caused by the desorption of the initially charged molecules. This is confirmed by the behavior of the work function in Figure 9b, which shows a concomitant strong decrease in this temperature range. This higher desorption temperature of the charged molecules relative to the neutral molecules can be expected due the electrostatic interaction of the charged molecule with its image charge. It may therefore seem surprising that the onset of the work function decrease and, therefore, the desorption of charged molecules happens already within the desorption regime of the neutral molecules. This might be explained by the spontaneous discharging as a result of the thermal energy distribution, enabling desorption as a neutral molecule.

## Conclusion

While charged 6P molecules could not be unambiguously identified by STM, valence band photoemission measurements allowed for their clear observation. Moreover, PT and ARUPS allowed the orientation and number of charged molecules to be quantified. The number of charged molecules and the determination if a given molecule was charged or not were found to be extremely sensitive to the precise work function of the particular MgO(100)/Ag(100) film preparation. This was investigated systematically by tuning the work function of a MgO(100)/Ag(100) film to values around the critical work function for integer charge transfer to 6P molecules. The simple relationship observed between the number of charged molecules and the work function change on the molecular adsorption proves that the charge transfer is governed by simple electrostatics. This confirms the conclusions drawn from a recent study with pentacene [20] and suggests that the ε_r_ of thin dielectrics may be significantly different from the values of their bulk counterparts (e.g., the ε_r_ of the MgO film presented here is approximately half the value of bulk MgO).

In contrast to pentacene, 6P is a geometrically flexible molecule. The charge transfer to 6P leads to both conformational and orientational changes and has a profound influence on the wetting and thermal stability of the 6P films. Upon anion formation, the molecule is planarized and orients exclusively along the [001] azimuth due to its electrostatic bond to the substrate. The presence of charged molecules hinders the mobility of the twisted neutral molecules [38]; therefore, when approximately 30% of the molecules are charged, a transition occurs and the neutral molecules also wet the surface. The conformational change induced by charge transfer leads to the final pinning work function being higher than the critical work function, as the latter is related to the electron affinity of the neutral twisted molecule, while the former is associated with the EA of the planar conformation. Increasing MgO thickness decreases the EA due to reduced polarization [20]. The Φ_crit_ is reduced to such a low value that systematic thickness-dependent studies of charge transfer to 6P could not be performed.

On dielectric interlayers, a simple measurement of the work function before and after the adsorbate overlayer growth is perhaps the most telling result with regard to electronic and physical coupling. If there is only a small reduction of Φ, then the system is in the vacuum level alignment regime and there is no charge transfer. If there is any significant change in Φ, then the system is in the Fermi level pinning regime with equilibrium achieved by a balance between charged and neutral adsorbates in the first monolayer. An increase in Φ, as in the examples here, implies a charge transfer from the underlying substrate to the LUMO while a decrease in Φ would indicate integer charge transfer from the HOMO to the substrate. The mode of charge transfer is primarily determined by the electron affinity and ionization potential levels and their relation to the Fermi level of the substrates. This is set by the dielectric/substrate work function.

It is important to emphasize that once in the Fermi level pinning regime, both charged and neutral species will co-exist on the dielectric. This has not been recognized until now because many of the standard techniques cannot easily distinguish between charged and neutral species. For reasonably large molecules with delocalized frontier orbitals, the integer charge is spread over many atoms and the resulting chemical shift will be too small to be seen with X-ray photoelectron spectroscopy [20]. Similarly, the LEED technique will probably not be sensitive to the charge state per se, although differences in the molecular orientation and in the long-range order might occur. With a thorough analysis and molecular orbital resolution, it should be possible to distinguish them with scanning probe techniques. Generally, however, studies with orbital resolution tend to focus on very low coverages, where only charged species will be present.

## Experimental

All experiments were performed under ultrahigh vacuum (UHV) conditions with a base pressure of ≤3 × 10^−10^ mbar. The Ag(100) crystal was cleaned by cycles of Ar^+^ sputtering and annealing at 500 °C. MgO(100) films were grown by Mg evaporation in an oxygen environment. The Mg fluxes used were on the order of 1 Å/min as monitored by a quartz microbalance. The MgO deposition was done at a temperature of 270 °C and at an O_2_ pressure of 10^−6^ mbar, followed by slow cooling (approximately 2.5 °C/min). This is the accepted procedure that provides epitaxial MgO(100) films with high structural quality [19,34]. One ML of MgO is defined as a single atomic layer (i.e., half a unit cell of crystalline bulk MgO), corresponding to a thickness of 2.105 Å. Work functions, measured from the secondary electron cutoff in the photoemission, could be reduced by annealing in UHV or further Mg exposure while annealing. The work function could be increased by O_2_ exposure (5 × 10^−7^–2 × 10^−4^ mbar) at moderate temperatures. Monitoring the work function during this post-preparation allowed for a close control of this parameter. *para*-Sexiphenyl (6P) was deposited at a rate of 0.8–3 Å/min at RT. When the 6P exposure is given in Angstroms, this refers to the microbalance reading with a density of 1 g/cm^3^. After conducting the ARUPS measurements on 6P/MgO(100)/Ag(100), 6P was desorbed from the substrate by flashing to 250 °C. Then, the work function of the substrate was modified as desired for the next 6P preparation. This allowed the experiments to be performed on a MgO film with the exact same thickness but with different work functions.

The photoemission tomography experiments were conducted at the Metrology Light Source insertion device beamline of the Physikalisch-Technische Bundesanstalt using the toroidal electron spectrometer. The ARUPS experiments were performed with a small hemispherical sector electron analyzer mounted on a goniometer (VG-ADES400) with a He I discharge lamp as photon source. Details of the PT and ARUPS setups as well as the identification of molecular orbitals can be found in work by Hollerer et al. [14]. The intensity values of the molecular photoemission features were determined by subtracting the background, measured prior to the adsorption, from the molecules and determining the area of the peak measured in the direction of the maximum emission intensity. At the He I of 21.22 eV, these were at 50° and 45° with respect to the surface normal along the [001] azimuthal direction for the LUMO and the HOMO, respectively.

The wetting parameter quantifies the spectral changes reflecting the wetting of 6P. It is calculated from the intensity of the MgO valence band peak prior to/after 6P deposition (*I*_MgO_/*I*_MgO'_) and the corresponding intensities (*I*_6P_/*I*_6P’_) in the energy range (between 14 eV and 6 eV below *E*_F_, normal emission) where the emission of the 6P sigma-orbitals is found, as


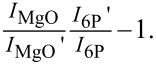


To assess the desorption dynamics, the samples were heated manually by a filament behind the crystal at a very low heating rate of approximately 0.1 °C/s (on average).

STM measurements were performed at −193 °C with a Createc low-temperature STM using electrochemically etched tungsten tips. The bias voltage was applied to the sample.
